# Supplementary material for: Globally distributed Myxococcota with photosynthesis gene clusters illuminate the origin and evolution of a potentially chimeric lifestyle
Source: Nat Commun. 2023 Oct 13;14:6450. doi: 10.1038/s41467-023-42193-7 (PMC10576062; doi:10.1038/s41467-023-42193-7)
Supplement: Supplementary file 1 — Supplementary Information [file 41467_2023_42193_MOESM1_ESM.pdf]

Supplementary Information for

Globally distributed *Myxococcota* with photosynthesis gene clusters illuminate the origin and evolution of a potentially chimeric lifestyle

Liuyang Li[1], Danyue Huang[2], Yaoxun Hu[1], Nicola M. Rudling[3], Daniel P. Canniffe[3], Fengping Wang[1,2*], Yinzhao Wang[1*]

[1]State Key Laboratory of Microbial Metabolism, School of Life Sciences and Biotechnology, Shanghai Jiao Tong University, Shanghai 200240, China

[2]School of Oceanography, Shanghai Jiao Tong University, Shanghai 200030, China

[3]Institute of Systems, Molecular and Integrative Biology, University of Liverpool, Liverpool, L69 7ZB, UK

*Fengping Wang and *Yinzhao Wang
Email: wyz@sjtu.edu.cn and fengpingw@sjtu.edu.cn.

Supplementary Note 1: Protologues

Supplementary Note 2: Sequences for Codon-optimised *Ca*. Kuafubacteriaceae *bchYZ* and *Nannocystaceae crtI*

Supplementary Figures include:

Figures S1 to S10

Supplementary Note 1: Protologues

Description of *Kuafubacterium* gen. nov

Kuafubacterium (Kua'fu.bac.te'ri.um. N.L. masc. n. *Kuafu* sun-chasing giant, Prometheus-like god in Chinese mythology; L. neut. n. *bacterium* a bacterium; *Kuafubacterium* a microbe associated with phototrophy).

The type species is *Kuafubacterium phototrophica*.

Description of *Kuafubacterium phototrophica* sp. nov

Kuafubacterium phototrophica (pho.to'tro.phi.ca. Gr. n. *phos* photos light; Gr. adj. *trophikos* nursing, tending or feeding; N.L. fem. adj. *phototrophica* referring to the likely capacity to use light for energy generation).

The nomenclatural type for the species is the genomic assembly LLY-WYZ-17_1 (GCA_016703535.1). Genome of LLY-WYZ-17_1 is predicted to 9.76 Mb in 2 scaffolds. The GC content is 66.46%. Genome has complete bacteriochlorophyll synthesis pathways, and encodes reaction center proteins and other key enzymes, suggesting potential phototrophic lifestyle. Genome also has key enzymes of CBB cycle, suggesting potential autotrophic capacity. Genome for this species originated from activated sludge.

Description of *Kuafucaenimonas* gen. nov

Kuafucaenimonas (Kua'fu.cae.ni.mo'nas. N.L. masc. n. *Kuafu* sun-chasing giant, Prometheus-like god in Chinese mythology; L. neut. n. *caenum* mud, sludge; L. fem. n. *monas* a monad; *Kuafucaenimonas* a microbe found in sludge environments associated with phototrophy).

The type species is *Kuafucaenimonas phototrophica*.

Description of *Kuafucaenimonas phototrophica* sp. nov

Kuafucaenimonas phototrophica (pho.to'tro.phi.ca. Gr. n. *phos* photos light; Gr. adj. *trophikos* nursing, tending or feeding; N.L. fem. adj. *phototrophica* referring to the likely capacity to use light for energy generation).

The nomenclatural type for the species is the genomic assembly LLY-WYZ-18_1 (GCA_020430745.1). Genome of LLY-WYZ-18_1 is predicted to 9.21 Mb in 1,562 scaffolds. The GC content is 71.29%. Genome has complete bacteriochlorophyll synthesis pathways, and encodes reaction center proteins and other key enzymes, suggesting potential phototrophic lifestyle. Genome also has key enzymes of CBB cycle, suggesting potential autotrophic capacity. Genome for this species originated from activated sludge.

Description of *Kuafuhalomonas* gen. nov

Kuafuhalomonas (Kua'fu.ha.lo.mo'nas. N.L. masc. n. *Kuafu* sun-chasing giant, Prometheus-like god in Chinese mythology; Gr. fem. n. *hals*, sea, saline, salt; L. fem. n. *monas* a monad; *Kuafuhalomonas* a microbe found in salt environments associated with phototrophy).
The type species is *Kuafuhalomonas phototrophica*.

Description of *Kuafuhalomonas phototrophica* sp. nov

Kuafuhalomonas phototrophica (pho.to'tro.phi.ca. Gr. n. *phos* photos light; Gr. adj. *trophikos* nursing, tending or feeding; N.L. fem. adj. *phototrophica* referring to the likely capacity to use light for energy generation).
The nomenclatural type for the species is the genomic assembly LLY-WYZ-16_1 (GCA_031800475.1). Genome of LLY-WYZ-16_1 is predicted to 5.30 Mb in 386 scaffolds. The GC content is 66.71%. Genome has complete bacteriochlorophyll synthesis pathways, and encodes reaction center proteins and other key enzymes, suggesting potential phototrophic lifestyle. Genome also has PR and *blh*, suggesting potential capacity using both proton-pumping and bacteriochlorophyll-based photosystems. Genome for this species originated from salt lagoon.

Description of *Kuafubacteriaceae* fam. nov

Kuafubacteriaceae (Kua'fu.bac.te.ra.ce'ae. N.L. masc. n. *Kuafubacterium* type genus of the family; L. suff. -*aceae*, ending to denote a family; N.L. fem. pl. n. *Kuafubacteriaceae* the family of the genus *Kuafubacterium*).
The description is the same as that for the genus *Kuafubacterium*. The type genus is *Kuafubacterium*. The family belongs to the order *Kuafubacteriales*.

Description of *Kuafubacteriales* ord. nov

Kuafubacteriales (Kua'fu.bac.te.ri.a'les. N.L. masc. n. *Kuafubacterium* type genus of the order; L. suff. -*ales*, ending to denote an order; N.L. fem. pl. n. *Kuafubacteriales* the order of the family *Kuafubacteriaceae*).
The description is the same as that for the genus *Kuafubacterium*. The type genus is *Kuafubacterium*. The order belongs to the class *Kuafubacteria*.

Description of *Kuafubacteria* class. nov

Kuafubacteria (Kua'fu.bac.te.ri.a. N.L. masc. n. *Kuafubacterium* type genus of the type order of the class; L. suff. -*ia* ending to denote a class; N.L. neut. pl. n. *Kuafubacteria* the class of the order *Kuafubacteriales*).
The description is the same as that for the genus *Kuafubacterium*. The type genus is *Kuafubacterium*. The class belongs to the phylum *Myxococcota*.

Description of *Houyibacterium* gen. nov

Houyibacterium ('Hou.yi.bac.te'ri.um. N.L. masc. n. *Houyi* Sun-shooting archer in Chinese mythology; L. neut. n. *bacterium* a bacterium; *Houyibacterium* a microbe associated with phototrophy).

The type species is *Houyibacterium oceanica*.

Description of *Houyibacterium oceanica* sp. nov

Houyibacterium oceanica (o.ce.a'ni.ca, N.L. fem. adj. *oceanica* of or pertaining to the ocean). The nomenclatural type for the species is the genomic assembly LLY-WYZ-15_3 (GCA_031800675.1). Genome is predicted to 9.41 Mb in 214 scaffolds. The GC content is 73.94%. Genome has complete bacteriochlorophyll synthesis pathways, and encodes reaction center proteins and other key enzymes, suggesting potential phototrophic lifestyle. Genome also has PR, suggesting the potential capacity using both proton-pumping and bacteriochlorophyll-based photosystems. Genomic assemblies for this species originated from seawater.

Description of *Houyihalomonas* gen. nov

Houyihalomonas ('Hou.yi.ha.lo.mo'nas. N.L. masc. n. *Houyi* Sun-shooting archer in Chinese mythology; Gr. fem. n. *hals* sea, saline, salt; *monas* L. fem. n. a monad; *Houyihalomonas* a microbe found in salt environments associated with phototrophy).

The type species is *Houyihalomonas phototrophica*.

Description of *Houyihalomonas phototrophica* sp. nov

Houyihalomonas phototrophica (pho.to'tro.phi.ca. Gr. n. *phos* photos light; Gr. adj. *trophikos* nursing, tending or feeding; N.L. fem. adj. *phototrophica* referring to the likely capacity to use light for energy generation).

The nomenclatural type for the species is the genomic assembly LLY-WYZ-14_1 (GCA_031800515.1). Genome is predicted to 4.70 Mb in 1,083 scaffolds. The GC content is 71.78%. Genome has complete bacteriochlorophyll synthesis pathways, and encodes reaction center proteins and other key enzymes, suggesting potential phototrophic lifestyle. Genome for this species originated from salt lagoon.

Description of *Houyibacteriaceae* fam. nov

Houyibacteriaceae ('Hou.yi.bac.te.ra.ce'ae. N.L. masc. n. *Houyibacterium* type genus of the family; L. suff. -*aceae*, ending to denote a family; N.L. fem. pl. n. *Houyibacteriaceae* the family of the genus *Houyibacterium*).

The description is the same as that for the genus *Houyibacterium*. The type genus is *Houyibacterium*. The family belongs to the order *Polyangiales*.

Description of *Xihepedomonas* gen. nov

Xihepedomonas (Xi.he.pe.do.mo'nas. N.L. fem. n. *Xihe* sun goddess in Chinese mythology; Gr. neut. n. *pedon* soil; L. fem. n. *monas* a monad; *Xihepedomonas* a microbe found in soil environments associated with phototrophy).

The type species is *Xihepedomonas phototrophica*.

Description of *Xihepedomonas phototrophica* sp. nov

Xihepedomonas phototrophica (pho.to'tro.phi.ca. Gr. n. *phos* photos light; Gr. adj. *trophikos* nursing, tending or feeding; N.L. fem. adj. *phototrophica* referring to the likely capacity to use light for energy generation).

The nomenclatural type for the species is the genomic assembly LLY-WYZ-3_1 (3300025461_5). Genome is predicted to 4.56 Mb in 324 scaffolds. The GC content is 70.81%. Genome has complete bacteriochlorophyll synthesis pathways, and encodes reaction center proteins and other key enzymes, suggesting potential phototrophic lifestyle. Genome also has key enzymes of CBB cycle, suggesting potential autotrophic capacity. Genome for this species originated from Arctic peat soil.

Description of *Xihelimnomonas* gen. nov

Xihelimnomonas (Xi.he.lim.no.mo'nas. N.L. fem. n. *Xihe* sun goddess in Chinese mythology; Gr. fem. n. *limne* a lake; L. fem. n. *monas* a monad; *Xihelimnomonas* a microbe found in lakes associated with phototrophy).

The type species is *Xihelimnomonas phototrophica*.

Description of *Xihelimnomonas phototrophica* sp. nov

Xihelimnomonas phototrophica (pho.to'tro.phi.ca. Gr. n. *phos* photos light; Gr. adj. *trophikos* nursing, tending or feeding; N.L. fem. adj. *phototrophica* referring to the likely capacity to use light for energy generation).

The nomenclatural type for the species is the genomic assembly LLY-WYZ-1_1 (3300027902_26). Genome is predicted to 3.47 Mb in 550 scaffolds. The GC content is 69.66%. Genome has complete bacteriochlorophyll synthesis pathways, and encodes reaction center proteins and other key enzymes, suggesting potential phototrophic lifestyle. Genome for this species originated from freshwater lake.

Description of *Xihecaenimonas* gen. nov

Xihecaenimonas (Xi.he.cae’ni.mo’nas. N.L. fem. n. *Xihe* sun goddess in Chinese mythology; L. neut. n. *caenum* mud, sludge; L. fem. n. *monas* a monad; *Xihecaenimonas* a microbe found in sludge environments associated with phototrophy).
The type species is *Xihecaenimonas phototrophica*.

Description of *Xihecaenimonas phototrophica* sp. nov
Xihecaenimonas phototrophica (pho.to’tro.phi.ca. Gr. n. *phos* photos light; Gr. adj. *trophikos* nursing, tending or feeding; N.L. fem. adj. *phototrophica* referring to the likely capacity to use light for energy generation).
The nomenclatural type for the species is the genomic assembly LLY-WYZ-6_1 (GCA_016793725.1). Genome is predicted to 9.75 Mb in 939 scaffolds. The GC content is 69.60%. Genome has complete bacteriochlorophyll synthesis pathways, and encodes reaction center proteins and other key enzymes, suggesting potential phototrophic lifestyle. Genome for this species originated from activated sludge.

Description of *Xihemicrobium* gen. nov
Xihemicrobium (Xi.he.mi.cro’bi.um. N.L. fem. n. *Xihe* sun goddess in Chinese mythology; N.L. neut. n. *microbium* (from Gr. adj. *mikros* small; and Gr. masc. n. *bios* life) a microbe; *Xihemicrobium* a microbe associated with phototrophy).
The type species is *Xihemicrobium phototrophica*.

Description of *Xihemicrobium phototrophica* sp. nov
Xihemicrobium phototrophica (pho.to’tro.phi.ca. Gr. n. *phos* photos light; Gr. adj. *trophikos* nursing, tending or feeding; N.L. fem. adj. *phototrophica* referring to the likely capacity to use light for energy generation).
The nomenclatural type for the species is the genomic assembly LLY-WYZ-5_1 (GCA_022843845.1). Genome is predicted to 4.95 Mb in 54 scaffolds. The GC content is 67.74%. Genome has complete bacteriochlorophyll synthesis pathways, and encodes reaction center proteins and other key enzymes, suggesting potential phototrophic lifestyle. Genome for this species originated from drinking water treatment.

Description of *Xihemicrobium aquatica* sp. nov
Xihemicrobium aquatica (a.qua’ti.ca. L. fem. adj. *aquatica* living, growing, or found in the water, aquatic).
The nomenclatural type for the species is the genomic assembly LLY-WYZ-4_1 (GCA_020247085.1). Genome is predicted to 7.36 Mb in 641 scaffolds. The GC content is 72.03%. Genome has complete bacteriochlorophyll synthesis pathways, and encodes reaction center proteins and other key enzymes, suggesting potential phototrophic lifestyle. Genomic assemblies for this species originated from freshwater lake.

Description of *Xihelimnobacterium* gen. nov

Xihelimnobacterium (Xi.he.lim.no.bac.te'ri.um. N.L. fem. n. *Xihe* sun goddess in Chinese mythology; Gr. fem. n. *limne* a lake; L. neut. n. *bacterium* a bacterium; *Xihelacuibacterium* a microbe found in lakes associated with phototrophy).

The type species is *Xihelimnobacterium phototrophica*.

Description of *Xihelimnobacterium phototrophica* sp. nov

Xihelimnobacterium phototrophica (pho.to'tro.phi.ca. Gr. n. *phos* photos light; Gr. adj. *trophikos* nursing, tending or feeding; N.L. fem. adj. *phototrophica* referring to the likely capacity to use light for energy generation).

The nomenclatural type for the species is the genomic assembly LLY-WYZ-2_2 (GCA_903911905.1). Genome is predicted to 7.57 Mb in 907 scaffolds. The GC content is 69.08%. Genome has complete bacteriochlorophyll synthesis pathways, and encodes reaction center proteins and other key enzymes, suggesting potential phototrophic lifestyle. Genomic assemblies for this species originated from freshwater lake.

Description of *Xihebacterium* gen. nov

Xihebacterium (Xi.he.bac.te'ri.um. N.L. fem. n. *Xihe* sun goddess in Chinese mythology; L. neut. n. *bacterium* a bacterium; *Xihebacterium* a microbe associated with phototrophy).

The type species is *Xihebacterium phototrophica*.

Description of *Xihebacterium phototrophica* sp. nov

Xihebacterium phototrophica (pho.to'tro.phi.ca. Gr. n. *phos* photos light; Gr. adj. *trophikos* nursing, tending or feeding; N.L. fem. adj. *phototrophica* referring to the likely capacity to use light for energy generation).

The nomenclatural type for the species is the genomic assembly LLY-WYZ-10_1 (GCA_016709225.1). Genome is predicted to 9.61 Mb in 12 scaffolds. The GC content is 71.15%. Genome has complete bacteriochlorophyll synthesis pathways, and encodes reaction center proteins and other key enzymes, suggesting potential phototrophic lifestyle. Genomic assemblies for this species originated from activated sludge.

Description of *Xihebacterium glacialis* sp. nov

Xihebacterium glacialis (gla.ci.a'lis. L. masc. adj. *glacialis* icy, frozen, full of ice, referring to the frozen, icy environment from which the species was found).

The nomenclatural type for the species is the genomic assembly LLY-WYZ-12_1 (GCA_014380655.1). Genome of LLY-WYZ-12_1 is predicted to 7.53 Mb in 1,963 scaffolds. The GC content is 69.41%. Genome has complete bacteriochlorophyll synthesis pathways, and encodes reaction center proteins and other key enzymes, suggesting potential phototrophic

lifestyle. Genome also contains a complete CBB cycle, suggesting potential photosynthetic capacity. LLY-WYZ-12_1 is found in glacier surface soil.

Description of *Xihebacterium aquatica* sp. nov

Xihebacterium aquatica (a.qua'ti.ca. L. fem. adj. *aquatica* living, growing, or found in the water, aquatic).

The nomenclatural type for the species is the genomic assembly LLY-WYZ-11_1 (GCA_001464385.1). Genome is predicted to 10.24 Mb in 400 scaffolds. The GC content is 72.20%. Genome has complete bacteriochlorophyll synthesis pathways, and encodes reaction center proteins and other key enzymes, suggesting potential phototrophic lifestyle. Genome for this species originated from drinking water treatment.

Description of *Xihecaenibacterium* gen. nov

Xihecaenibacterium (Xi.he.cae'ni.bac.te'ri.um. N.L. fem. n. *Xihe* sun goddess in Chinese mythology; L. neut. n. *caenum* mud, sludge; L. neut. n. *bacterium* a bacterium; *Xihecaenibacterium* a microbe found in sludge environments associated with phototrophy).

The type species is *Xihecaenibacterium phototrophica*.

Description of *Xihecaenibacterium phototrophica* sp. nov

Xihecaenibacterium phototrophica (pho.to'tro.phi.ca. Gr. n. *phos* photos light; Gr. adj. *trophikos* nursing, tending or feeding; N.L. fem. adj. *phototrophica* referring to the likely capacity to use light for energy generation).

The nomenclatural type for the species is the genomic assembly LLY-WYZ-9_1 (GCA_016794345.1). Genome is predicted to 9.66 Mb in 321 scaffolds. The GC content is 69.69%. Genome has complete bacteriochlorophyll synthesis pathways, and encodes reaction center proteins and other key enzymes, suggesting potential phototrophic lifestyle. Genome for this species originated from activated sludge.

Description of *Xihemonas* gen. nov

Xihemonas (Xi.he.mo'nas. N.L. fem. n. *Xihe* sun goddess in Chinese mythology; L. fem. n. *monas* a monad; *Xihemonas* a microbe associated with phototrophy).

The type species is *Xihemonas phototrophica*.

Description of *Xihemonas phototrophica* sp. nov

Xihemonas phototrophica (pho.to'tro.phi.ca. Gr. n. *phos* photos light; Gr. adj. *trophikos* nursing, tending or feeding; N.L. fem. adj. *phototrophica* referring to the likely capacity to use light for energy generation).

The nomenclatural type for the species is the genomic assembly LLY-WYZ-8_1 (GCA_022842465.1). Genome is predicted to 9.57 Mb in 431 scaffolds. The GC content is

71.38%. Genome has complete bacteriochlorophyll synthesis pathways, and encodes reaction center proteins and other key enzymes, suggesting potential phototrophic lifestyle. Genome for this species originated from drinking water treatment.

Description of *Xihemonas sinensis* sp. nov

Xihemonas sinensis (si.nen'sis. N.L. fem. adj. *sinensis* pertaining to China, referring to the geographical location of the species).

The nomenclatural type for the species is the genomic assembly LLY-WYZ-7_1 (GCA_018266155.1). Genome is predicted to 10.87 Mb in 159 scaffolds. The GC content is 70.03%. Genome has complete bacteriochlorophyll synthesis pathways, and encodes reaction center proteins and other key enzymes, suggesting potential phototrophic lifestyle. Genome for this species originated from activated sludge.

Description of *Xihehalomonas* gen. nov

Xihehalomonas (Xi.he.ha.lo.mo'nas. N.L. fem. n. *Xihe* sun goddess in Chinese mythology; Gr. fem. n. *hals* sea, saline, salt; L. fem. n. *monas* a monad; *Xihehalomonas* a microbe found in salt environments associated with phototrophy).

The type species is *Xihehalomonas phototrophica*.

Description of *Xihehalomonas phototrophica* sp. nov

Xihehalomonas phototrophica (pho.to'tro.phi.ca. Gr. n. *phos* photos light; Gr. adj. *trophikos* nursing, tending or feeding; N.L. fem. adj. *phototrophica* referring to the likely capacity to use light for energy generation).

The nomenclatural type for the species is the genomic assembly LLY-WYZ-13_1 (GCA_031800425.1). Genome is predicted to 7.61 Mb in 542 scaffolds. The GC content is 73.72%. Genome has complete bacteriochlorophyll synthesis pathways, and encodes reaction center proteins and other key enzymes, suggesting potential phototrophic lifestyle. Genome for this species originated from salt lagoon.

Supplementary Note 2: Sequences for Codon-optimised *Ca*. Kuafubacteriaceae *bchYZ* and *Nannocystaceae crtI*

Codon-optimised *Ca*. Kuafubacteriaceae *bchYZ*

AGATCTATGACGCGTGAGGGACCTTCCTGCTCGGGCGATAAACGCAAACTTCGTGAGGCAGCCGCGAAGGCAGGG
GCGGGGGAGACCCTTGAACAATACGCACAGGACTATCCCATGGGTCCGCACGATCAACCGCAGAGCATGTGCCCGG
CGTTCGGTTCGCTCCGTGTGGGGCTCCGCATGCGCCGCACGGCGACAATCCTCTCGGGAAGCGCCTGCTGCGTCT
ATGGTCTGACGTTCACGTCGCATTTCTACGGCGCGCGTCGCACTGTCGGTTATGTGCCGTTCGATTCGGAGAGCCTT
GTGACCGGGAAACTGTACGAGGATATTCGCGAAGCCGTGCACGAGATGGCAGACCCGGAACATTATGACGCGATCG
TGGTTACCAATCTCTGTGTGCCGACGGCCTCCGGTGTCCCGCTGCGTCTGCTTCCGAAAGAAATTGATGGCGTGCGT
ATCATCGGCATTGATGTGCCCGGCTTCGGTGTCCCCACGCACGCGGAAGCCAAAGACGTGCTGGCGGGAGCCATG
CTGCAATATGCACGCCAGGAGGCCGAACAAGGCCCCGTCGCCGCCCCCCGCGGAGGCCCGTCGGAGCGTCCGAC
CGTCACCCTGGTCGGTGAGATGTTCCCGGTGGACCCTGTGACCATTGGCCGCATGCTTGAGCCTCTCGGGCTGGCC
GCGGGACCGGTGGTCCCGACCCGCGAATGGCGCGAATTGTATGCGGCGCTTGATGGCGCGGTGGTCGCCGGGATT
CATCCGTTTTATACGGCGGCCTTTCGTGAATTTTCGGCCGCGGGGCGCCCGATTGTCGGCTCGGCGCCGGTCGGGC
TGGATGGGACCGCGACGTGGCTGGATGAAATTTCGAAAGCCTGTGGCGTCCCTCAACAGAAAGTCGATGCGGCCAA
AGCCCAACTGCTTCCGGCCATTCGCTCCGCCCTCGATGCCGCGCCTATCAAAGGTCGCATCACCCTGTCGGGGTAT
GAAGGGTCGGAACTTATCGTGGCCCGCCTCCTGATCGAAAGCGGGGCCGATGTCCGTTATGTCGGCACGGCGTGTC
CGCGTACCGAATGGTCGGATCCGGATCGCGAATGGCTGGAAGCGCGCGGGGTGCAGGTCCGCTATCGCGCCTCGC
TTGAACAAGATATCCAGGCGATGGAAGAATTCAAACCGGATCTGACCGTCGGGACCACGCCCGTGGTCCAGGCCGC
GAAAGAACGTGCCATCCCGTCGCTGTATTTCACCAATCTGATTAGCGCCCGGCCGCTCATGGGCCCGGCGGGCGCG
GGGTCGCTTGCCCAAGTGATCAATGCGGCCTTGGGCTCCAAAAATCGCTTCGATGAAATGCGCGAATTCTTCGAAGG
CGTCGGCCAGGGTTTCAATGCGGGGGTGTGGGAACAGGTTCCGGTCGATCGTCCCAAATTCCGCGAACAACAGCTC
GTGCAGCTCCGGCGCCATAAGCGAAAGCTGAAGAAGGAGGAGGCGGTCAAATGCTAGTGCTGGATCATGATCGGGC
CGGCGGCTATTGGGGGGCGGTTTATGTGTTCACCAGCGTCAAAGGGCTGCAGGTCATTATTGATGGGCCGGTCGGC
TGTGAAAATCTGCCCGTGACCAGCGTCCTGCATTATACGGATGGCCTTCCCCCTCATGAGTTGCCGGTGTGTGTCAC
GGGGCTGGCAGAAGAGGAACTCGGGCAGAAAGGCACGGAAGGGGCCATGCATCGGGCGCATGAATATCTCGAGGA
TAGCCTCCCGTCCGTCGTCGTAACCGGGTCGATTGCGGAAATGATTGGCGGTGGGGTGACCCCTGAAGGCACGGG
CATAATGCGGTTCCTCCCTCGCACCATCGATGAGGATCAGTGGCAGTCGGCGAATCGGGCGATGAATTGGATTTGG
GAACAGTGGGGCGCCAAGAAAGGGCGCATGCCTAAACCCCGGCCCAAAAAAGACGGTGATAAACCGAAGGTCAATA
TCATCGGCCCGTCGTATGGGTATTTTAATACCTGGTCCGATCTCGCAGAAATCCGCAGGCTTATTGAAGGCATCGGA
GCTGAAGTGCATATGGTGTTCCCCCTCGGGACCCATCTGGCGGATGTCGAACGTCTGGTCGACGCAGAAGTGAACG
TGTGTATGTATCGCGAATACGGCCGTCGCCTTTGTGAAGCGCTGGATCGACCCTATCTGCAAGCGCCGATCGGCCT
GCATTCGACCACCAAGTTTCTGCGCAAACTGGGGGAACTCTTGGGGCTGGATCCCGAACCGTTCATTGAAAAAGAAA
AACATACCACGATCAAACCTATCTGGGATCTCTGGCGCAGCGTCACGCAGGATTTCTTCGGGACCGCATCGTTTGCC
GTCGTTGCGAATGAAACCTATACGCGCGGGCTGCGCAATTATCTGGAAACCGAACTGGGGCTGCCCTGTACCTTTAG
CTTCAGCCGCTCGGCCGGCCGTAAACCGGATAATGCGACCGTCCGGCAGCATGTCCGCGAAAAAACGCCGCTGATT
ATGTTCGGGTCGTATAATGAACGCATGTATCTGGCCGAAGCCGGCTCGAAAGCGATGTATATCCCGGCCTCCTTCCC
GGGCACCGCGATCCGCCGCCATACGGGCACCCCTTTCATGGGCTATGCCGGGGCCACGTATATTCTCCAAGAAGTT
TGTAATGCCCTGTTCGATGCCCTTTTCCATATCCTGCCTCTGGGCACCGATATGGATGCTGTCGATGCCACCCCCTC
GCGTCTTCATGAAGAACTCCCGTGGGATGATGAAGCCCAACGGGAACTGGATAAACTGATGGAAAAAGTCCCGGTG
CTGACGCGCATCTCCGCCGCGAAAATGCTGCGGGATGCATCCGAACGGGAAGCCAAACGCCTTGGAGAAGAACGC
GTTACCGCCCAACGGGTCGCCGCTACCCATGCAGAAACCGCCAAAGGCTGGGCCGCGTGAACTAGT

BglII-*bchY*-*bchZ*-SpeI

Codon-optimised *Nannocystaceae crtI*

AGATCTATGCAAACCGAACCCCGCGATCGCGATCCTCGTTCCAGGCCCCATGCCATCGTCATCGGTAGCGGCTTTG
GAGGCCTCGCGGCGGCGGTTCGCCTCGGCGCCCGCGGATATGATGTGACCGTGCTTGAACGCCGCGATCGCCCCG
GCGGTCGCGCCTATGTCTATCAACAAGATGGCTTTACCTTCGACGGCGGGCCGACCGTCATCACGGCGCCGTTCCT
GTTTGAAGAACTGTGGACGCTGTGTGGTCGTCGGCTGCAAGATGATGTCGAACTGCTTCCGGTGACCCCGTTCTATC
GCATCCGCTTCGATGATGGCGCGCTGTTCGATTATACCGGCGATCCCGAAGCCATGCGTGCCGAAGTGCAGCGGCT
CGCGCCCGAAGATGTCGATGGCTATGAACGCTTCCTCGATATGAGCCGGCGCATCTATGAAGTCGGCTTCGAACAG
CTCGCCCATGTGCCGTTCGATCGCTGGACCGATATGGCGCGCATCGTGCCGGAAATGATGCGGCTGCGCAGCTATC
GCACCGTCTATGGGCTTGTCTCGTCGTTTGTGCGCAATGATCGACTGCGGCAGATCCTCTCGTTCCATCCGCTGTTG
GTGGGCGGCAATCCGTTCGAAACCACCTCGATCTATTCGCTCATCTGTCATCTCGAACGTCGCTTCGGTGTGTGGTT
CCCCAAAGGCGGCACGGGGGCGCTGGTCGACGGCCTCGTGCGGCTCATCGAAGGCCAAGGCAATCGCGTTCGCTG
TAATGTGGAAGTCTCGCAGATCCATGTGGTCGATGGTGCCGCGCGCGGCGTCGTGCTCGCCGATGGCTCGCGGATC
GATGCCGATGTGGTTGTGTCGAATGCCGATGCCGGCTGGACCTATAAACATCTGTTGCCGGCCAGCGCGCGCAAAC
ATTGGACCGATGCGCGGGTCGAAAAAGCCCGCTATTCGATGAGCCTGTTCGTGTGGTATTTCGGCACCAATCGACGC
TATGAAGACGTCGCGCATCACACCATCATGCTCGGGCCCCGCTATAAACCCCTGCTCGATGACATCTTCAAACATAA
GCGGCTCGCACCCGATTTCAGCCTCTATCTGCATCGGCCGACCGCCAGCGATCCCTCGCTGGCGCCCGCGGGGCA
TGATGGCTTCTATGTGCTCTCGCCGGTGCCGCATCTCGATGCCGATGTCGATTGGACCGTGGAAACCGAACCCTATC
GCCGCAAACTCGAACAGTTCCTCGATCAGACCATCTTGCCGGGGCTGTCGCAATCGTTGGTGAGCTCGCGGACGCT
CACGCCGCTGGGATTCCGCGATGATCTGTTGTCGCTCAAAGGCGCGGCCTTCGGCATGGAACCGGTGCTCCTGCAG
TCGGCGTTCTTCCGCCCGCACAATCGCAGCGAAGATATCTCGGGACTGTATCTGGTCGGCGCCGGCACGCATCCGG
GTGCGGGCGTGCCGGGGGTGCTGTCTTCGGCGCGCGTGCTGGATCGCGTGGTGCCCTCGGCGCAAGAAGCACTTG
CCGCGCGTCGCGAACCCGCGGTTGTCGCGTCGCTTGAAGCACGTCATGATGCCGCCTAAACTAGT

BglII-*crtI*-SpeI

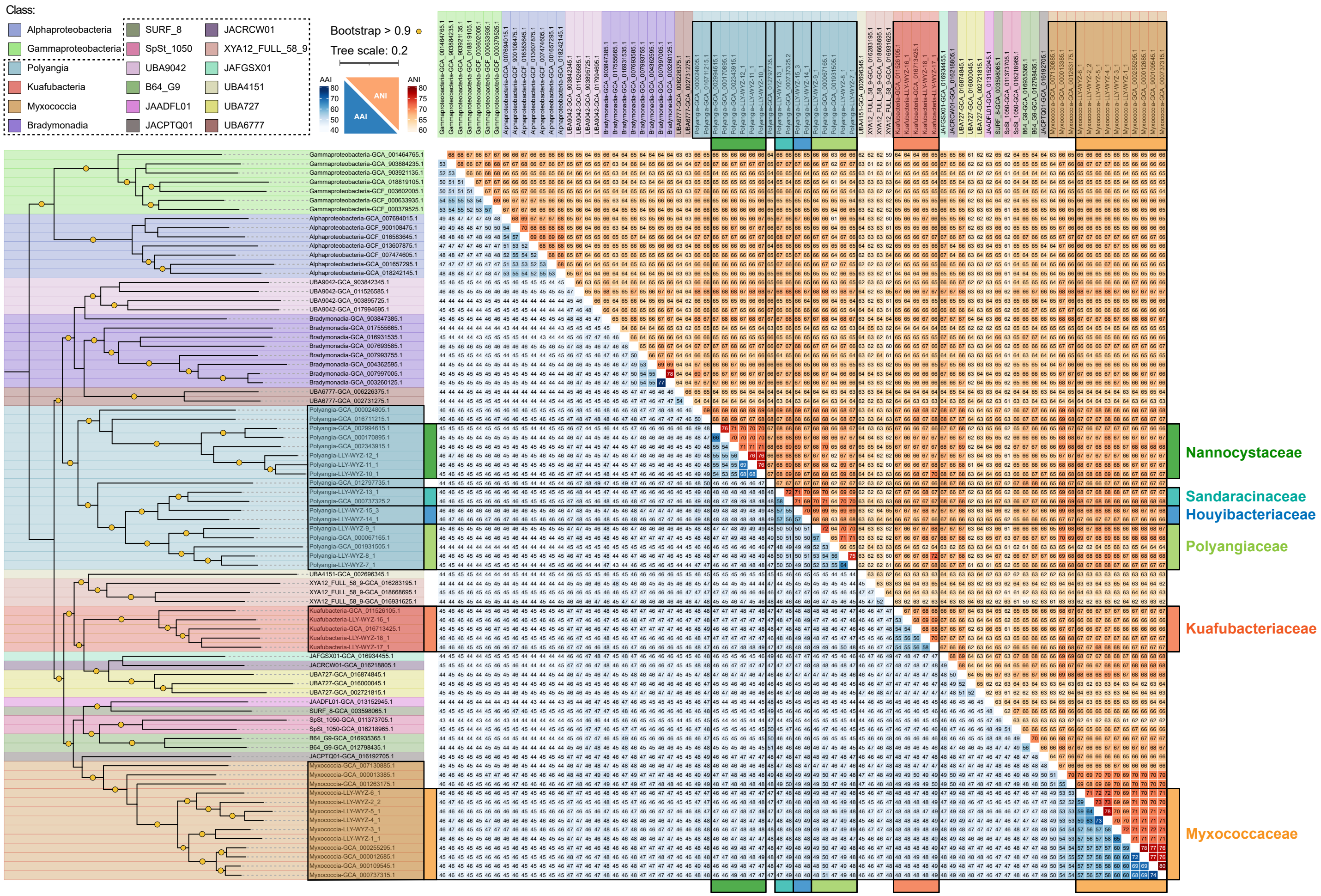

Figure S1 Phylogenetic affiliations and paired relationships of average nucleotide identities (ANIs) and average amino acid identities (AAIs) of representative MAGs of *Myxococcota*. Phylogenomic affiliation of the MAGs based on the concatenated alignments of 120 conserved single-copy marker genes from GTDB-Tk. The tree was built using FastTree with 1,000 bootstrap replicates. The bootstrap supporting values above 0.9 are indicated with solid circles. The phylogenetic tree was rooted by Proteobacteria. All classes of *Myxococcota* and *Proteobacteria* are assigned different background colors. Labels of *Polyangia, Ca.* Kuafubacteria*, and Myxococcia* are highlighted. Six potential phototrophic families of *Myxococcota* are assigned different strip colors. Detailed information for representative MAGs is provided in Supplementary Data 4.

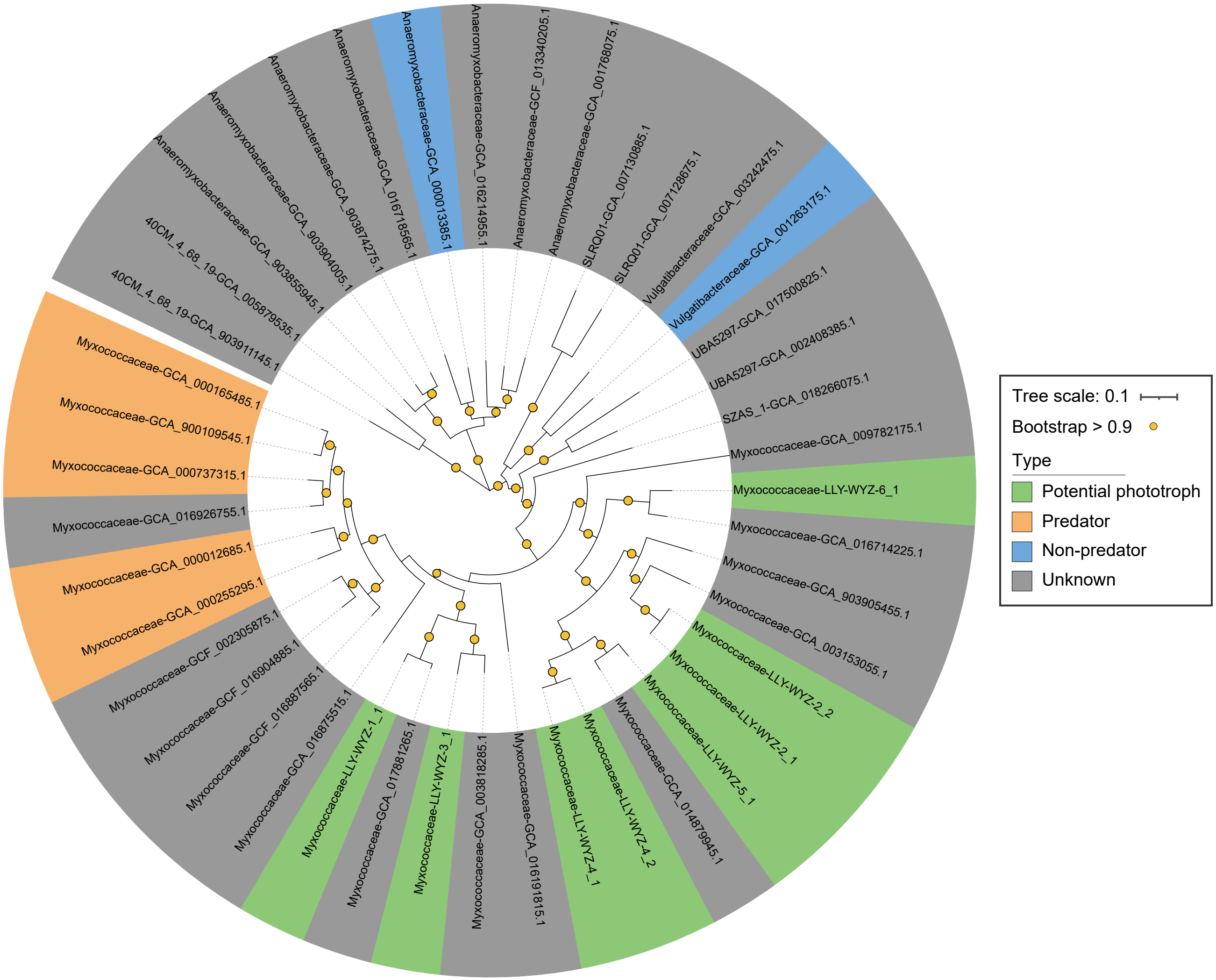

Figure S2 Phylogenetic affiliations of representative MAGs of the class *Myxococcia* from *Myxococcota*. Phylogenomic affiliation of the MAGs based on the concatenated alignments of 120 conserved single-copy marker genes from GTDB-Tk. The tree was built using FastTree with 1,000 bootstrap replicates. The bootstrap supporting values above 0.9 are indicated with solid circles. Potential phototrophic genomes are highlighted with green background. Predatory genomes are highlighted with orange background. Non-Predatory genomes are highlighted with blue background. Reference MAGs without potential phototrophic and/or varified predatory capacity are highlighted with grey bacground. Detailed information for representative MAGs is provided in Supplementary Data 5.

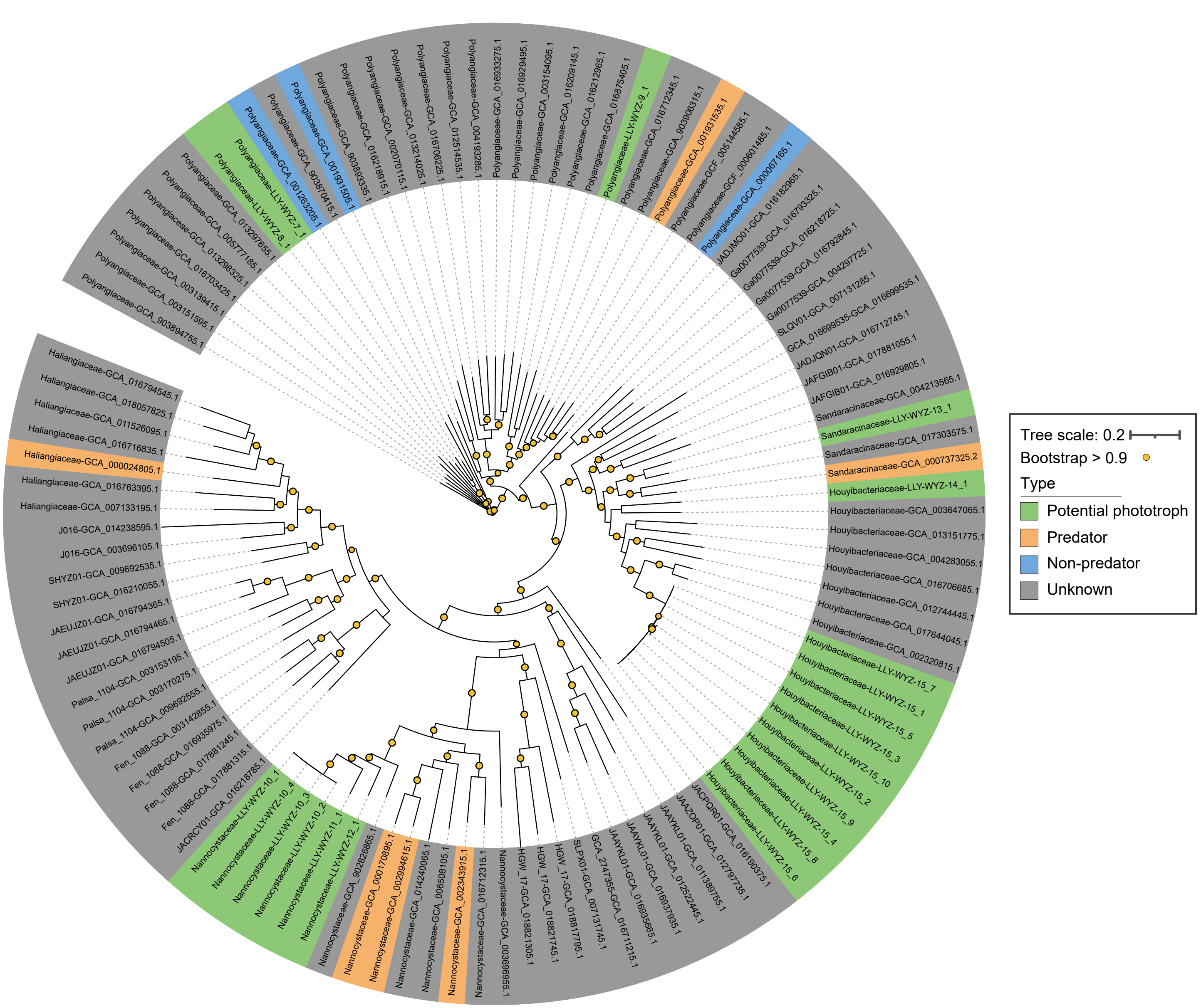

Figure S3 Phylogenetic affiliations of representative MAGs of the class *Polyangia* from *Myxococcota*. Phylogenomic affiliation of the MAGs based on the concatenated alignments of 120 conserved single-copy marker genes from GTDB-Tk. The tree was built using FastTree with 1,000 bootstrap replicates. The bootstrap supporting values above 0.9 are indicated with solid circles. Potential phototrophic genomes are highlighted with green background. Predatory genomes are highlighted with orange background. Non-Predatory genomes are highlighted with blue background. Reference MAGs without potential phototrophic and/or varified predatory capacity are highlighted with grey bacground. Detailed information for representative MAGs is provided in Supplementary Data 5.

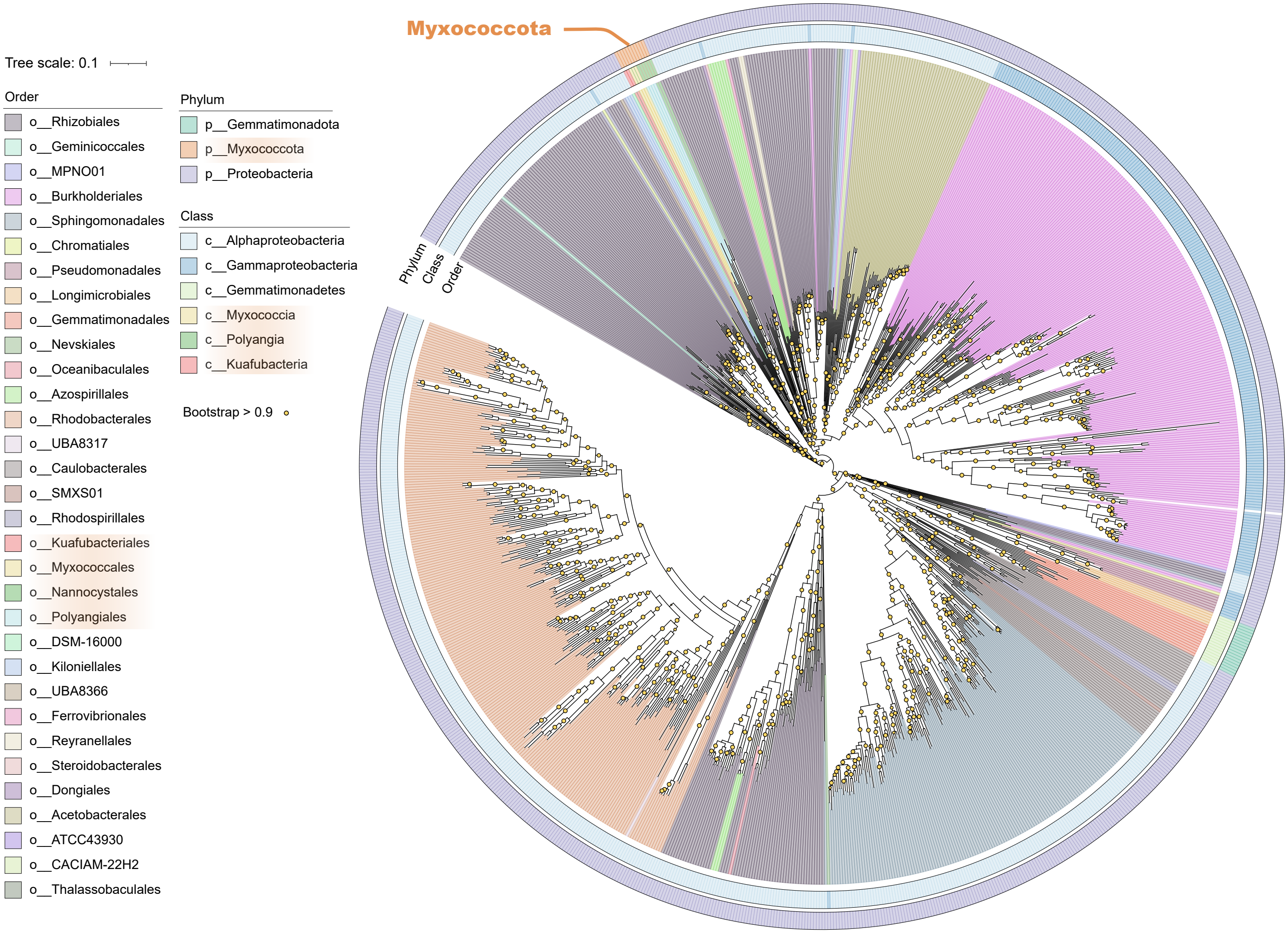

Figure S4 Phylogenetic affiliations of the PGCs. The phylogenetic tree with a total of 973 PGCs was constructed based on the alignments of conservative PGC proteins occurring in at least 70% of non-redundant genomes. Alignments were based on MAFFT and then filtered with trimAl, and the tree was built using FastTree with parameter -wag with a bootstrap value of 1,000. The bootstrap supporting values above 0.9 are indicated with solid circles. The tree is colored by the order-level, the inner ring is colored based on the class-level, and the outer ring was colored based on the phylum-level.

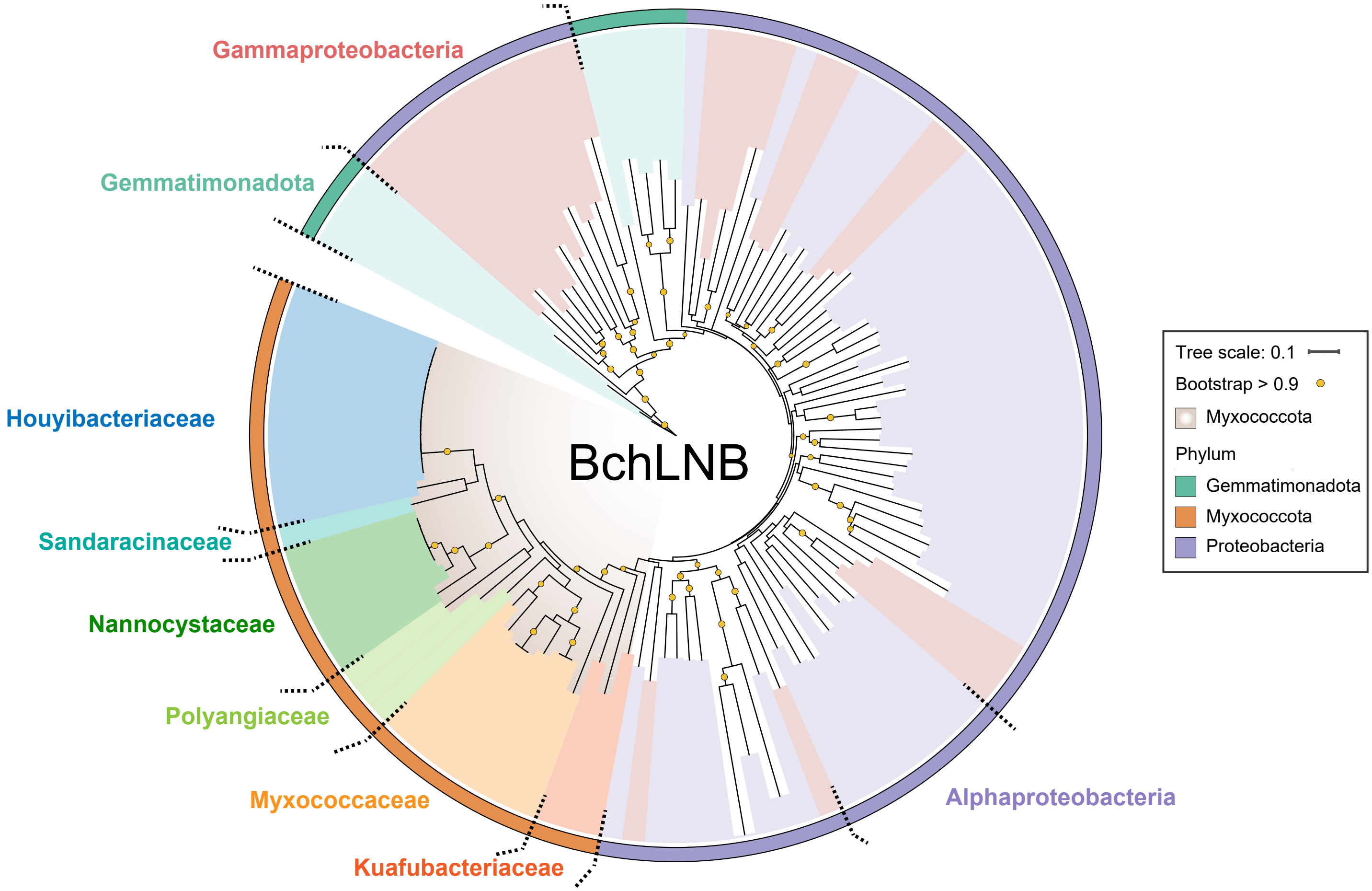

Figure S5 Phylogenetic affiliations of the BchLNB. The phylogenetic tree was constructed based on the alignments of BchLNB with 1,203 aligned positions. Alignments were based on MAFFT and then filtered with trimAl, and the tree was built using IQ-Tree with 1,000 bootstrap replicates. The bootstrap supporting values above 0.9 are indicated with solid circles. Lineages of *Myxococcota* are colored with brown gradient for background. Members from *Alphaproteobacteria*, *Gammaproteobacteria* (including *Betaproteobacteria*), *Gemmatimonadota*, and 6 families of *Myxococcota* were assigned different background colors. The outer ring of phylogeny is colored based on corresponding phylum. Six potential phototrophic families of *Myxococcota* are also labeled near their phylogenies.

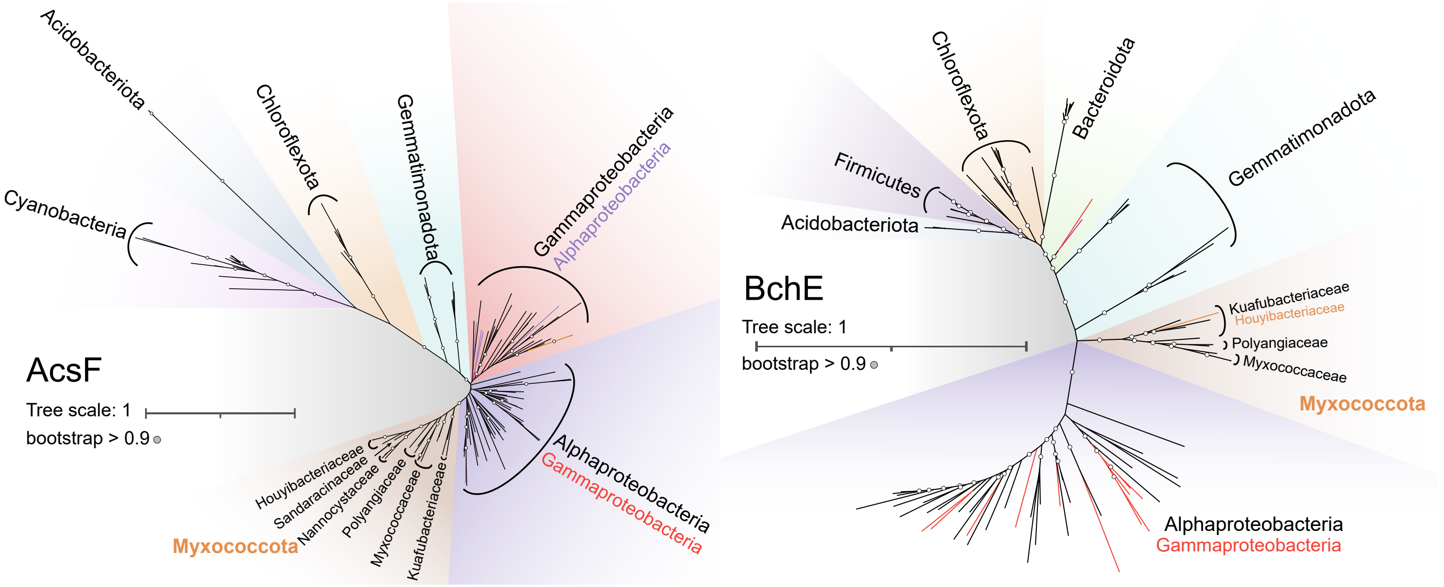

Figure S6 Phylogenetic affiliations of the AcsF and BchE. The phylogenetic trees were constructed based on the alignments of AcsF with 282 aligned positions and BchE with 498 aligned positions. Alignments were based on MAFFT and then filtered with trimAl, and the trees were built using IQ-Tree with the models LG+R5+C60 and LG+R6+C60 with 1,000 bootstrap replicates, respectively. The bootstrap supporting values above 0.9 were indicated with solid circles. Lineages of *Myxococcota*, *Alphaproteobacteria*, *Gammaproteobacteria* (including *Betaproteobacteria*), *Gemmatimonadota*, *Chloroflexota*, *Acidobacteriota*, *Firmicutes*, *Bacteroidota*, and *Cyanobacteria* were assigned different background colors. Six potential phototrophic families of *Myxococcota* are also labeled near their phylogenies.

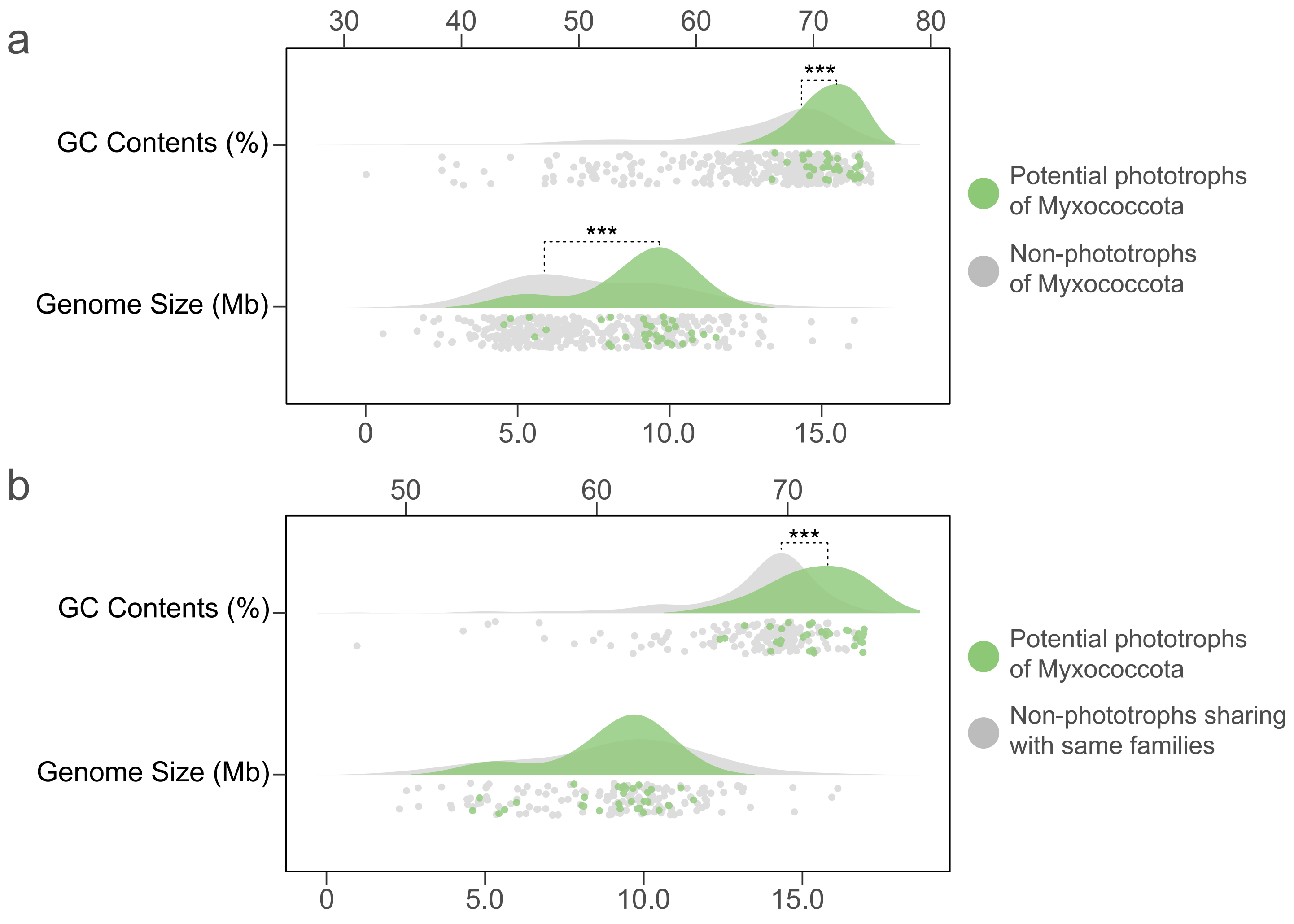

Figure S7 The comparisons of GC contents and genome sizes between potential phototrophic members and reference non-phototrophic members of *Myxococcota*. (A) The comparisons between potential phototrophic members and other members in the phylum. (B) The comparisons between potential phototrophs and non-phototrophs sharing with same families. Significant differences were determined by the nonparametric Wilcoxon rank-sum tests (***$P < 0.001$). Detailed information for reference genomes is provided in Supplementary Data 6. Source data are provided as a Source Data file.

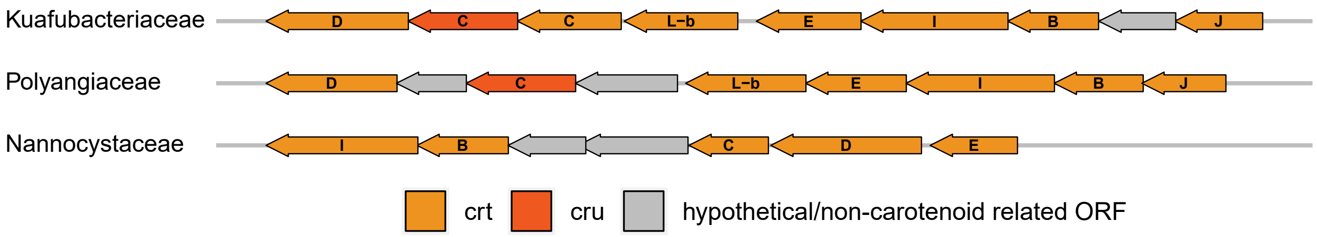

Figure S8 Gene organizations of representative carotenoid gene clusters. Carotenoid genes are colored: *crt* (orange) and *cru* (red), carotenoid biosynthesis genes; gray, hypothetical or non-carotenoid-related genes.

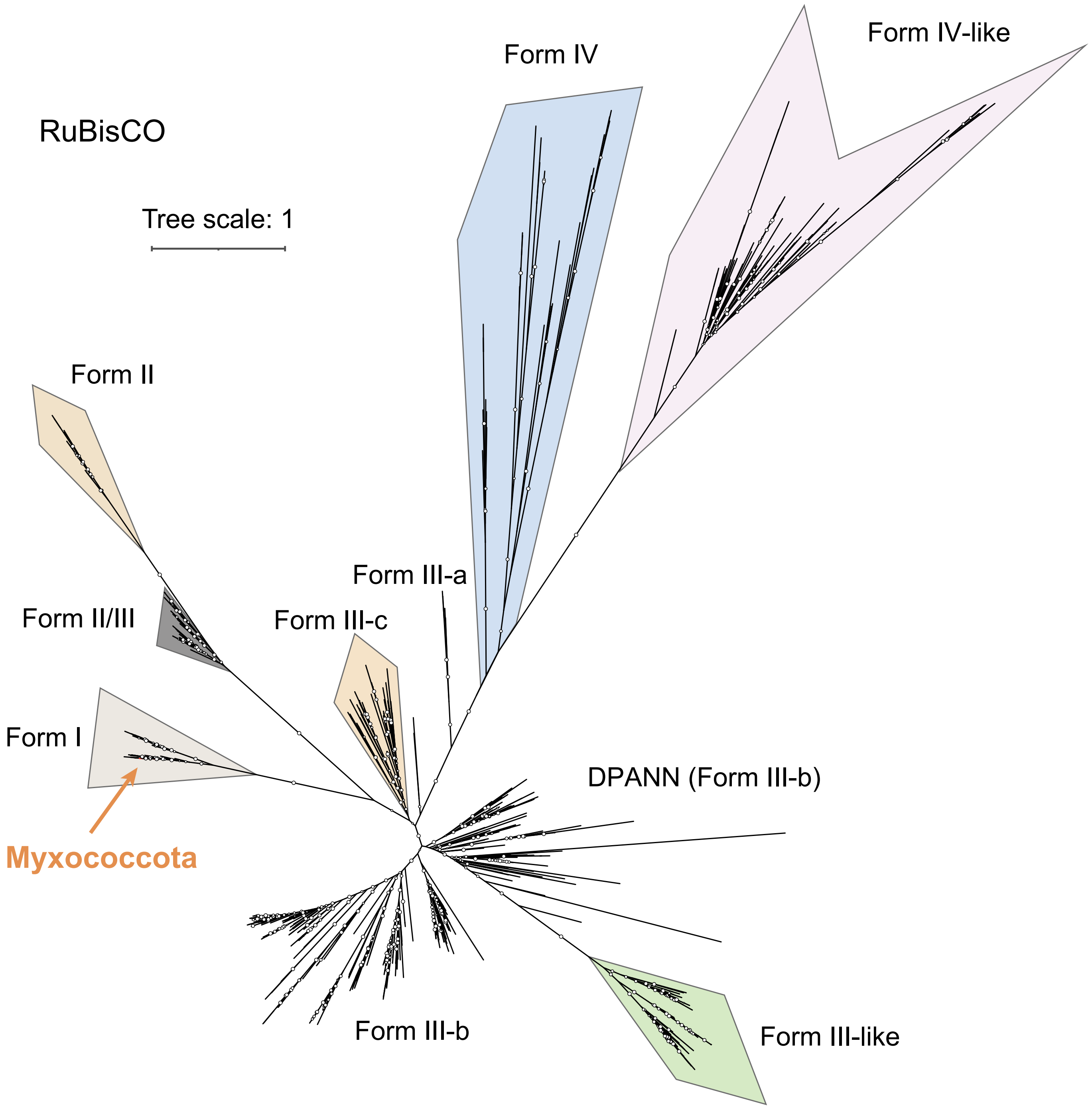

Figure S9 Phylogenetic affiliations of the RuBisCO. The reference protein sequences of RuBisCO were retrieved from the published paper (Alexander L. Jaffe et al., 2019). The phylogenetic tree was constructed based on the alignments of RuBisCO with 399 aligned positions. Alignments were based on MAFFT and then filtered with trimAl, and the trees were built using IQ-Tree with the models LG+R10+C60 with 1,000 bootstrap replicates. The bootstrap supporting values above 0.8 are indicated with solid circles.

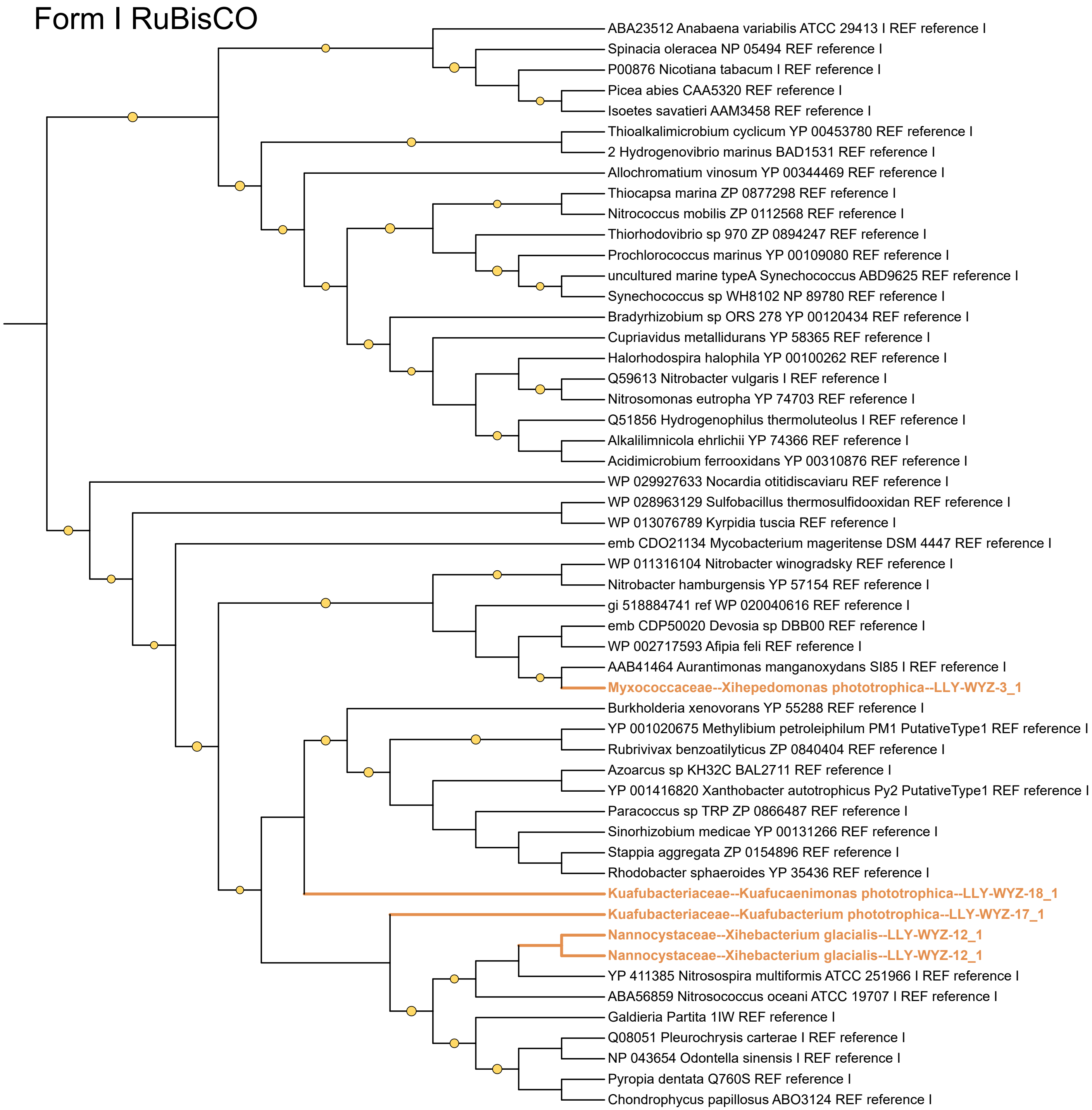

Figure S10 Phylogenetic affiliations of the form I clade of RuBisCO. The subtree was selected from the total RuBisCO tree. The bootstrap supporting values above 0.8 are indicated with solid circles. Lineages of *Myxococcota* are listed as bold font and colored with orange.
