# Supplementary material for: Globally distributed Myxococcota with photosynthesis gene clusters illuminate the origin and evolution of a potentially chimeric lifestyle
Source: Nat Commun. 2023 Oct 13;14:6450. doi: 10.1038/s41467-023-42193-7 (PMC10576062; doi:10.1038/s41467-023-42193-7)
Supplement: Supplementary file 3 — Description of Additional Supplementary Files [file 41467_2023_42193_MOESM3_ESM.pdf]

Description of Additional Supplementary Files:

Supplementary Data 1: The metadata, taxonomy, and statistics of 32 potential phototrophic Myxococcota genomes.

Supplementary Data 2: The functional gene BLASTP results of 32 potential phototrophic Myxococcota genomes, and selection of representative genomes.

Supplementary Data 3: The concatenated set of 37 marker genes used to construct species tree.

Supplementary Data 4: The genomes used in Figure S1.

Supplementary Data 5: The genomes used in Figure S2 and S3.

Supplementary Data 6: The GTDB taxonomy of reference genomes used in Figure S7.

Supplementary Data 7: The estimated relative abundances based on reads coverages of potential phototrophic Myxococcota and other genomes from the same sample.

Supplementary Data 8: Marker proteins used in the PGC trees.

Supplementary Data 9: The annotation results of potential phototrophic Myxococcota using eggNOG 5.0 database.

Supplementary Data 10: Models used in the phylogenetic trees.

Supplementary Data 11: Nomenclature and Etymology for the taxonomy of potential phototrophic Myxococcota.

Supplementary Data 12: Word roots used for automated nomenclature.
