# Supplementary material for: Globally distributed Myxococcota with photosynthesis gene clusters illuminate the origin and evolution of a potentially chimeric lifestyle
Source: Nat Commun. 2023 Oct 13;14:6450. doi: 10.1038/s41467-023-42193-7 (PMC10576062; doi:10.1038/s41467-023-42193-7)
Supplement: Supplementary file 5 — Reporting Summary [file 41467_2023_42193_MOESM5_ESM.pdf]

nature portfolio

Corresponding author(s): Fengping Wang and Yinzhao Wang

Last updated by author(s): Sep 17, 2023

Reporting Summary

Nature Portfolio wishes to improve the reproducibility of the work that we publish. This form provides structure for consistency and transparency in reporting. For further information on Nature Portfolio policies, see our Editorial Policies and the Editorial Policy Checklist.

Statistics

For all statistical analyses, confirm that the following items are present in the figure legend, table legend, main text, or Methods section.

n/a	Confirmed	
☐	☒	The exact sample size (*n*) for each experimental group/condition, given as a discrete number and unit of measurement
☐	☒	A statement on whether measurements were taken from distinct samples or whether the same sample was measured repeatedly
☐	☒	The statistical test(s) used AND whether they are one- or two-sided<br>*Only common tests should be described solely by name; describe more complex techniques in the Methods section.*
☒	☐	A description of all covariates tested
☒	☐	A description of any assumptions or corrections, such as tests of normality and adjustment for multiple comparisons
☐	☒	A full description of the statistical parameters including central tendency (e.g. means) or other basic estimates (e.g. regression coefficient) AND variation (e.g. standard deviation) or associated estimates of uncertainty (e.g. confidence intervals)
☐	☒	For null hypothesis testing, the test statistic (e.g. *F*, *t*, *r*) with confidence intervals, effect sizes, degrees of freedom and *P* value noted<br>*Give P values as exact values whenever suitable.*
☒	☐	For Bayesian analysis, information on the choice of priors and Markov chain Monte Carlo settings
☒	☐	For hierarchical and complex designs, identification of the appropriate level for tests and full reporting of outcomes
☒	☐	Estimates of effect sizes (e.g. Cohen's *d*, Pearson's *r*), indicating how they were calculated

Our web collection on statistics for biologists contains articles on many of the points above.

Software and code

Policy information about availability of computer code

Data collection	No software was used to collect data.
Data analysis	All scripts and softwares to perform metagenome assembly, annotation and binning can be accessed from github (https://github.com/) or are listed in methods with versions and can be downloaded from the websites provided in the publications in the references part in the manuscript. The specific links to the custom software are listed below: DIAMOND version 2.0.14.152: http://ab.inf.uni-tuebingen.de/software/diamond/, sickle version 1.33: https://github.com/najoshi/sickle, MEGAHIT version 1.2.9-hotfix1: https://hku-bal.github.io/megabox/, BBMap version 38.18: https://github.com/BioInfoTools/BBMap/, Prodigal version 2.6.3: http://compbio.ornl.gov/prodigal/, MetaBAT version 2:2.15: https://bitbucket.org/berkeleylab/metabat, CheckM version 1.1.3: http://ecogenomics.github.io/CheckM, compareM version 0.1.2: https://github.com/dparks1134/CompareM, OrthoANIu version 1.2: https://www.ezbiocloud.net/tools/orthoaniu.Keywords, MAFFT version 7.490: https://mafft.cbrc.jp/alignment/software/, trimAl version 1.4.1: http://trimal.cgenomics.org, fastp version 0.12.4: https://github.com/OpenGene/fastp, GTDB-Tk version 2.0.0: https://github.com/Ecogenomics/GTDBTk, dRep version 3.4.0: https://github.com/MrOlm/drep, CheckM2 version 1.0.0: https://github.com/chklovski/CheckM2, CoverM version 0.6.1: https://github.com/wwood/CoverM, FastTree version 2.1.11: http://www.microbesonline.org/fasttree, treePL: https://github.com/blackrim/treePL, MEGA11 version 11.0.13: https://www.megasoftware.net, IQ-Tree version 2.1.2: http://www.iqtree.org, salmon version 1.9.0: https://github.com/COMBINE-lab/salmon, Great Automatic Nomenclator (GAN): https://github.com/telatin/gan.

For manuscripts utilizing custom algorithms or software that are central to the research but not yet described in published literature, software must be made available to editors and reviewers. We strongly encourage code deposition in a community repository (e.g. GitHub). See the Nature Portfolio guidelines for submitting code & software for further information.

Data

Policy information about availability of data

All manuscripts must include a data availability statement. This statement should provide the following information, where applicable:

- Accession codes, unique identifiers, or web links for publicly available datasets
- A description of any restrictions on data availability
- For clinical datasets or third party data, please ensure that the statement adheres to our policy

The genomes of Myxococcota with photosynthesis genes generated in this study have been deposited in the NCBI GenBank under BioProject ID PRJNA943119, as well as the eLibrary of Microbial Systematics and Genomics (eLMSG; https://www.biosino.org/elmsg/index) under accession numbers LMSG_G000011443.1, LMSG_G000011444.1, LMSG_G000011445.1, LMSG_G000011446.1, LMSG_G000011447.1, LMSG_G000011448.1, LMSG_G000011449.1, LMSG_G000011450.1, LMSG_G000011451.1, LMSG_G000011452.1. The databases used in this study include NCBI database (https://www.ncbi.nlm.nih.gov/), GEM catalog (https://genome.jgi.doe.gov/portal/GEMs/GEMs.home.html), GTDB database Release 207 (https://data.gtdb.ecogenomic.org/releases/release207/), and KEGG database (https://www.genome.ad.jp/kegg/).

Human research participants

Policy information about studies involving human research participants and Sex and Gender in Research.

Reporting on sex and gender	N/A
Population characteristics	N/A
Recruitment	N/A
Ethics oversight	N/A

Note that full information on the approval of the study protocol must also be provided in the manuscript.

Field-specific reporting

Please select the one below that is the best fit for your research. If you are not sure, read the appropriate sections before making your selection.

☐ Life sciences ☐ Behavioural & social sciences ☒ Ecological, evolutionary & environmental sciences

For a reference copy of the document with all sections, see nature.com/documents/nr-reporting-summary-flat.pdf

Ecological, evolutionary & environmental sciences study design

All studies must disclose on these points even when the disclosure is negative.

Study description	Photosynthesis is the most fundamental biogeochemical process on Earth, known to be restricted to a few bacterial and eukaryotic phyla. Understanding the evolution of Earth's ecosystem depends largely on fully unraveling the origin and evolution of phototrophic organisms, which can be impeded and biased by the difficulties of cultivation. Here, we utilized meta-omics approaches to search for novel photosynthetic organisms, their ecological activities and evolution. We discovered novel phototrophic bacteria belonging to the phylum Myxococcota, which were known for their sociality, predation and the production of bioactive secondary metabolites. A photosynthesis gene cluster encoding a type II reaction center appears in at least six Myxococcota families from three classes, suggesting a vertical evolutionary history among these taxa. Photosynthesis genes were found actively expressed in various natural environments, indicating their previously overlooked roles in myxococcotal carbon and energy transformation. Heterologous expression of the myxococcotal pigment biosynthesis genes further demonstrate the capability of these organisms to grow as phototrophs. Overall, our results provide the first evidence of potential photosynthetic ability in Myxococcota, which would provide insights for the evolution of a potentially chimeric lifestyle in prokaryotes.
Research sample	No field sampling were performed in this study. Metagenomic and genomic datasets were collected online. In order to discover novel phototrophic prokaryotes, we collected and analyzed 2,194 publicly available shotgun metagenomes originated from hydrosphere-related environments, including surface seawaters, marine sediments, and freshwater niches, as well as 474,091 public available genomes from the NCBI prokaryotes database and GEM database. Thirty two high-quality MAGs, which represent nearly all publicly available genomes of potential phototrophic Myxococcota, were used for further analyses. The metagenomes are available in the NCBI repository at https://www.ncbi.nlm.nih.gov/. The accession numbers of the metagenomes and genomes that were used to analyze potential phototrophic Myxococcota are listed in Supplementary Data 1.
Sampling strategy	In the current study, sampling strategy is not applicable because we mainly focused on discovery of potential phototrophic predatory prokaryotes in natural environments. We screened MAGs binned from more than 2,000 metagenomic datasets from public available NCBI SRA database and nearly all public available genomes from NCBI prokaryotes database and GEM database.
Data collection	The first author Liuyang Li collected all datasets from the NCBI prokaryotes database and SRA repository and did the analyses.

	Liuyang Li, Danyue Huang, and Yaoxun Hu participated in collecting dataset of the GEM database and collecting metadata from the scientific literature .
Timing and spatial scale	The data collection started/end at July 2022. The MAGs of potential phototrophic Myxococcota were recovered globally from diverse habitats spanning large spatial scale, including the aquatic and terrestrial habitats, salt lagoon and freshwater lake, oligotrophic and eutrophic seawater, high- and low-latitude geographical zones, as well as anthropogenic ecosystems.
Data exclusions	No data were excluded from the analyses.
Reproducibility	Reproducibility is not relevant and it's straightforward to reproduce the findings according to the described methods.
Randomization	Randomization is not relevant since our study aims to discover potential phototrophic Myxococcota in natural environments. It is necessary to keep all high-quality MAGs of potential phototrophic Myxococcota, using all metagenomic data collected in this study.
Blinding	Blinding was not relevant to this study, given that it primarily involved the analyses of environmental metagenomic datasets that cannot be influenced by human manipulation.

Did the study involve field work? ☐ Yes ☒ No

Reporting for specific materials, systems and methods

We require information from authors about some types of materials, experimental systems and methods used in many studies. Here, indicate whether each material, system or method listed is relevant to your study. If you are not sure if a list item applies to your research, read the appropriate section before selecting a response.

Materials & experimental systems

n/a	Involved in the study
☒	☐ Antibodies
☒	☐ Eukaryotic cell lines
☒	☐ Palaeontology and archaeology
☒	☐ Animals and other organisms
☒	☐ Clinical data
☒	☐ Dual use research of concern

Methods

n/a	Involved in the study
☒	☐ ChIP-seq
☒	☐ Flow cytometry
☒	☐ MRI-based neuroimaging
